# Paediatric Pharmacovigilance: Use of Pharmacovigilance Data Mining Algorithms for Signal Detection in a Safety Dataset of a Paediatric Clinical Study Conducted in Seven African Countries

**DOI:** 10.1371/journal.pone.0096388

**Published:** 2014-05-01

**Authors:** Dan K. Kajungu, Annette Erhart, Ambrose Otau Talisuna, Quique Bassat, Corine Karema, Carolyn Nabasumba, Michael Nambozi, Halidou Tinto, Peter Kremsner, Martin Meremikwu, Umberto D’Alessandro, Niko Speybroeck

**Affiliations:** 1 Research Institute of Health and Society (IRSS), Université catholique de Louvain, Brussels, Belgium; 2 Institute of Tropical Medicine, Antwerp, Belgium; 3 Malaria Public Health Department, University of Oxford-KEMRI-Wellcome Trust Programme, Nairobi, Kenya; 4 Uganda Malaria Surveillance project/Infectious Disease Research Collaboration, Kampala, Uganda; 5 Centro de Investigação em Saúde de Manhiça (CISM), Maputo, Mozambique/Barcelona Centre for International Health Research (CRESIB, Hospital Clínic-Universitat de Barcelona), Barcelona, Spain; 6 National Malaria Control Program–TRAC Plus, Ministry of Health, Kigali, Rwanda; 7 Epicentre, Paris, France/Mbarara University of Science and Technology, Faculty of Medicine, Mbarara, Uganda; 8 Tropical Diseases Research Centre, Ndola, Zambia; 9 Institut de Recherches en Sciences de la Santé, Bobo Dioulasso, Burkina Faso/Centre Muraz, Bobo Dioulasso, Burkina Faso; 10 Department of Paediatrics, University of Calabar, Calabar, Nigeria/Institute of Tropical Diseases Research & Prevention, Calabar, Nigeria; 11 Medical Research Council Unit, Fajara, The Gambia; 12 Santé Stat. and Analytical Research Institute (SSARI), Kampala, Uganda; 13 Institut für Tropenmedizin, Universität Tübingen, Germany and Centre de Recherches Médicales de Lambaréné, Lambaréné, Gabon; Nottingham University, United Kingdom

## Abstract

**Background:**

Pharmacovigilance programmes monitor and help ensuring the safe use of medicines which is critical to the success of public health programmes. The commonest method used for discovering previously unknown safety risks is spontaneous notifications. In this study we examine the use of data mining algorithms to identify signals from adverse events reported in a phase IIIb/IV clinical trial evaluating the efficacy and safety of several Artemisinin-based combination therapies (ACTs) for treatment of uncomplicated malaria in African children.

**Methods:**

We used paediatric safety data from a multi-site, multi-country clinical study conducted in seven African countries (Burkina Faso, Gabon, Nigeria, Rwanda, Uganda, Zambia, and Mozambique). Each site compared three out of four ACTs, namely amodiaquine-artesunate (ASAQ), dihydroartemisinin-piperaquine (DHAPQ), artemether-lumefantrine (AL) or chlorproguanil/dapsone and artesunate (CD+A). We examine two pharmacovigilance signal detection methods, namely proportional reporting ratio and Bayesian Confidence Propagation Neural Network on the clinical safety dataset.

**Results:**

Among the 4,116 children (6–59 months old) enrolled and followed up for 28 days post treatment, a total of 6,238 adverse events were reported resulting into 346 drug-event combinations. Nine signals were generated both by proportional reporting ratio and Bayesian Confidence Propagation Neural Network. A review of the manufacturer package leaflets, an online Multi-Drug Symptom/Interaction Checker (DoubleCheckMD) and further by therapeutic area experts reduced the number of signals to five. The ranking of some drug-adverse reaction pairs on the basis of their signal index differed between the two methods.

**Conclusions:**

Our two data mining methods were equally able to generate suspected signals using the pooled safety data from a phase IIIb/IV clinical trial. This analysis demonstrated the possibility of utilising clinical studies safety data for key pharmacovigilance activities like signal detection and evaluation. This approach can be applied to complement the spontaneous reporting systems which are limited by under reporting.

## Background

Historically, there have been several examples of patients being harmed by prescribed marketed medicines, the thalidomide tragedy being the paradigmatic case [1]. Today decisions to prescribe and administer medications are influenced by the associated risks of adverse drug reactions. Although adult patients are aware of possible risks of adverse effects related to treatments, for similarly exposed children, the risk is weighed by a parent or guardian who can subsequently provide the necessary consent. An adverse drug reaction (ADR) is defined as any harm associated with the use of given medications at a normal dosage during normal use. ADR may occur following a single dose or prolonged administration of a drug or result from the combination of two or more drugs [2]. An ADR is different from an adverse event (AE) which has been defined as any untoward medical occurrence in a patient or clinical investigation subject to whom a pharmaceutical product has been administered and which does not necessarily have a causal relationship with the treatment. An AE can therefore be any unfavourable and unintended sign (that could include a clinically significant abnormal laboratory finding), symptom or disease temporally associated with the use of a medicinal product, whether or not considered related to the medicinal product.

Pharmacovigilance has been defined as the process of evaluating and improving the safety of marketed medicines [3]. The WHO defines ‘pharmacovigilance’ as “the science and activities relating to the detection, assessment, understanding and prevention of adverse effects or any other drug related problems” [4]. Some rare ADRs are not identified during pre-marketing clinical trials, rather after the treatment has been marketed and a relatively large number of patients have been exposed to it. A drug safety signal is defined as information that arises from one or multiple sources (including observations and experiments), which suggests a new potentially causal association, or a new aspect of a known association, between an intervention and an event or a set of related events, either adverse or beneficial, that is judged to be of sufficient likelihood to justify verification [5]. In pharmacovigilance, signal detection refers primarily to the generation and preliminary assessment of hypotheses suited to explain any relevant safety observation. Traditionally, such an analysis is conducted by a systematic manual review of every report sent by physicians to pharmacovigilance experts.

Post-marketing surveillance analyses are based on ADR notifications that are voluntarily submitted to the national pharmacovigilance centres by healthcare providers, industry-sponsored phase IV clinical trials, or through prospective clinical registries [6]. Since the 1960s, this spontaneous reporting system has been the mainstay for generating drug safety data in some countries but this practice has only been introduced in Africa in the last decade [7]. The system relies heavily on the manufacturer, consumers and healthcare providers to identify and report ADRs, and therefore may be limited by under reporting [7]. Clinical trials and other controlled studies usually provide more complete and homogeneously compiled data which are submitted to the regulatory authorities and presented in standard formats. Indeed, all recruited patients comply with specific inclusion and exclusion, are well described, and are regularly monitored by the research team to uncover any ADRs. Efficacy and safety analysis are usually well documented and carefully controlled.

The analytical methods used in post-marketing pharmacovigilance to analyse ADR data and generate suspected ADR signals include biostatistics and data mining algorithms (DMA) [8]. Data mining is the technique of extracting hidden associations or patterns of association in large datasets when manual inspection is not feasible. The most commonly used data mining methods in pharmacovigilance include proportional reporting ratio (PRR) [9], a Bayesian confidence propagation neural network (BCPNN) [10], and the multi-item gamma Poisson shrinker (MGPS) [11], [12]. All of these are based on a quantitative measure of disproportionality between observed and expected reports of a certain drug-event combination when comparing with all other reports of adverse events and drugs in the dataset. Each of these data mining methods derives a different statistical measure to qualify the ratio of observed-to-expected reports, consequently generating a signal indicative of a safety problem. The PRR has been used on the European Medicines Agency EudraVigilance database, the BCPNN by the Uppsala Monitoring Centre on the WHO safety database, and the MGPS on the database of the Food and Drug Administration (FDA) [13].

The aim of this study was to examine the feasibility and usefulness of two DMAs to generate and identify signals exploiting the safety database of a large multi-country phase IIIb/IV clinical study.

## Methods

### Data Source

This analysis utilised data from a multi-centric phase IIIb/IV clinical study that was conducted between July 2007 and July 2009, in twelve sites located in seven African countries (Burkina Faso, Gabon, Nigeria, Rwanda, Uganda, Zambia, Mozambique) [14]. Each site compared three of the four artemisinin-based combination therapies (ACTs) for treatment of uncomplicated malaria i.e. amodiaquine-artesunate (ASAQ), dihydroartemisinin-piperaquine (DHAPQ), artemether-lumefantrine (AL) or chlorproguanil/dapsone and artesunate (CD+A). In each site, one of the three ACTs was already approved as first line treatment for uncomplicated malaria and was already being utilised in the respective populations. Throughout the study period, safety was assessed through both direct observation using physical exam, vital signs and laboratory tests by the clinician and the caretaker reporting an event to the clinic after the patient has been discharged. Adverse events (AEs) and serious adverse events (SAEs) reports were coded according to the system organ class (SOC) code, high level term (HLT), preferred term (PT) using the MedDRA (Medical Dictionary for Regulatory Activities) [15]; other variables in the safety database included treatment given to patient, and date of onset of the AE.

### Signal Detection

The two methods applied were the proportional reporting ratio (PRR) [9], and the Bayesian confidence propagation neural network (BCPNN) [10] – a frequentist and a Bayesian based framework, respectively. Classically, pharmacovigilance data mining methods are formulated with contingency table structured databases [16]. Each spontaneous report may involve several suspected drugs and several observed events, leading to *J* (total number of) drugs and *I* (total number of) events mentioned at least once in a report (Table 1).

**Table 1:** A two by two table for the adverse event-drug pair.

	Reports with drug of interest, *j*	Reports of all other drugs in database	Total
Reports with AE*of interest, *i*	*a*	*b_._*	*a+b*
Reports of all other AEs in database	*c*	*d*	*c+d*
**Total**	*a+c*	*b+d*	*a+b+c+d*

*AE = Adverse Event; *a = *the number of reports involving the drug of interest *j* and adverse event of interest *i* combination; *b = *reports of adverse event of interest *i* observed with other drugs; *c = *reports of all other AEs with drug *j; d = *reports of all other AEs with the other drugs; and *a+b+c+d* = the total number of reports in the dataset.

The suspected AE signals were generated using the R software [17] by applying PhViD package [18] which is a data mining package containing several pharmacovigilance signal detection methods extended to the multiple comparison setting.

### Signals Evaluation

We adopted a common pharmacovigilance practice [19], [13] where the clinical validity of the drug-event associations are identified by the proposed method and reviewed by an expert in the therapeutic area. The signal detection and evaluation was done following procedures described elsewhere [20]. Briefly, signals were evaluated in two stages, first by comparing the generated signals with the already known drug-events relationship using the manufacturer package leaflets; secondly, they were submitted to a clinician with experience on malaria treatments. The drug manufacturer package leaflets for DHAPQ, AL and ASAQ were used to eliminate ADRs already mentioned onto the leaflet. The Multi-Drug Symptom/Interaction Checker software DoubleCheckMD (http://doublecheckmd.com) – was also used for AL since it is the only ACT that was found in that database. The frequency of the generated but known signals was compared with the frequency already reported on the leaflet to establish whether this difference deserved further evaluation. The unknown signals were submitted to four clinicians to identify signals considered to be rare events deserving further analysis. These clinicians eliminated events which were most likely due to the disease and those known to be caused by other concomitant medications. If two signals were thought to be related, a sensitivity analysis was done, by removing one of the two and subsequently rerunning the model. If both signals did not appear again, they were considered as correlated and thus treated as a single signal, otherwise they were treated independently. Finally, these signals were ranked using the signal indices produced by each method.

### Ethical Considerations and Patients Safety

The clinical trial [14] was registered prior to the enrollment of the first patient in the ClinicalTrials.gov registry (NCT00393679, http://clinicaltrial.gov/ct2/show/NCT00393679) and in the Pan African Clinical Trials Registry. (/PACTR2009010000911750, http://www.pactr.org/). Permission to use the related safety dataset were obtained from all site investigators of the study team.

## Results

A total of 6,238 adverse events were reported within the 28 days of follow up after taking any of the four ACTs resulting into a total of 346 drug-AE combinations. The severity or any perceived relationship with the drug was not taken into account in this analysis, and multiple events were often reported after treatment with a drug. From the 346 drug-AE combinations, the two DMAs generated nine suspected signals which were subjected to review by the experts (Table 2). Drug-AE combinations involving ASAQ had more signals (n = 5) than the other ACTs while DHAPQ had only one.

**Table 2:** A Comparison of suspected signals detected by the two data mining algorithms.

Drug	Method and suspected signal events		Number of reports
	BCPNN	PRR	
**AL**	Eye discharge	Eye discharge	12
	Malaria	Malaria	79
	Pyrexia	Pyrexia	411
**ASAQ**	ALT increased	ALT increased	12
	Anaemia	Anaemia	168
	Bronchopneumonia	Bronchopneumonia	12
	Neutrophil count increased	Neutrophil count increased	12
	WBC count decreased	WBC count decreased	8
**DHAPQ**	Haemorrhagic diarrhoea	Haemorrhagic diarrhoea	9

AL = Artemether-Lumefantrine; ASAQ = Artesunate-Amodiaquine; DHAPQ = Dihydroartemisinin- Piperaquine; WBC = White Blood Cells; ALT = Alanine amino Transferase.

A third of the generated signals (three out of nine) were already listed on the manufacturer package leaflets and in the DoubleCheckMD. Comparing the frequency of such reports in our database with the information in the leaflets, the signal ASAQ-Anemia was reported for about one in five patients in our database making it more frequent compared to the one in 100 patients indicated onto the leaflet. Similarly, the signal AL-Pyrexia was reported by one third of the patients which is slightly more frequent than in the leaflet (greater than 1 in every 10 patients treated). After review, two signals, i.e. AL-Malaria and AL-Pyrexia, were eliminated as they were due to the underlying disease. The remaining five signals were as follows: i) ‘AL-Eye discharge’; ii)‘ASAQ-increased Alanine amino Transferase (ALT)’; iii) ‘ASAQ-Bronchopneumonia’; iv) ‘ASAQ-Neutrophil count increased’; and v) DHAPQ-“haemorrhagic diarrhoea”, and resulted in the new hypotheses that may need further evaluation.

**Figure 1:**
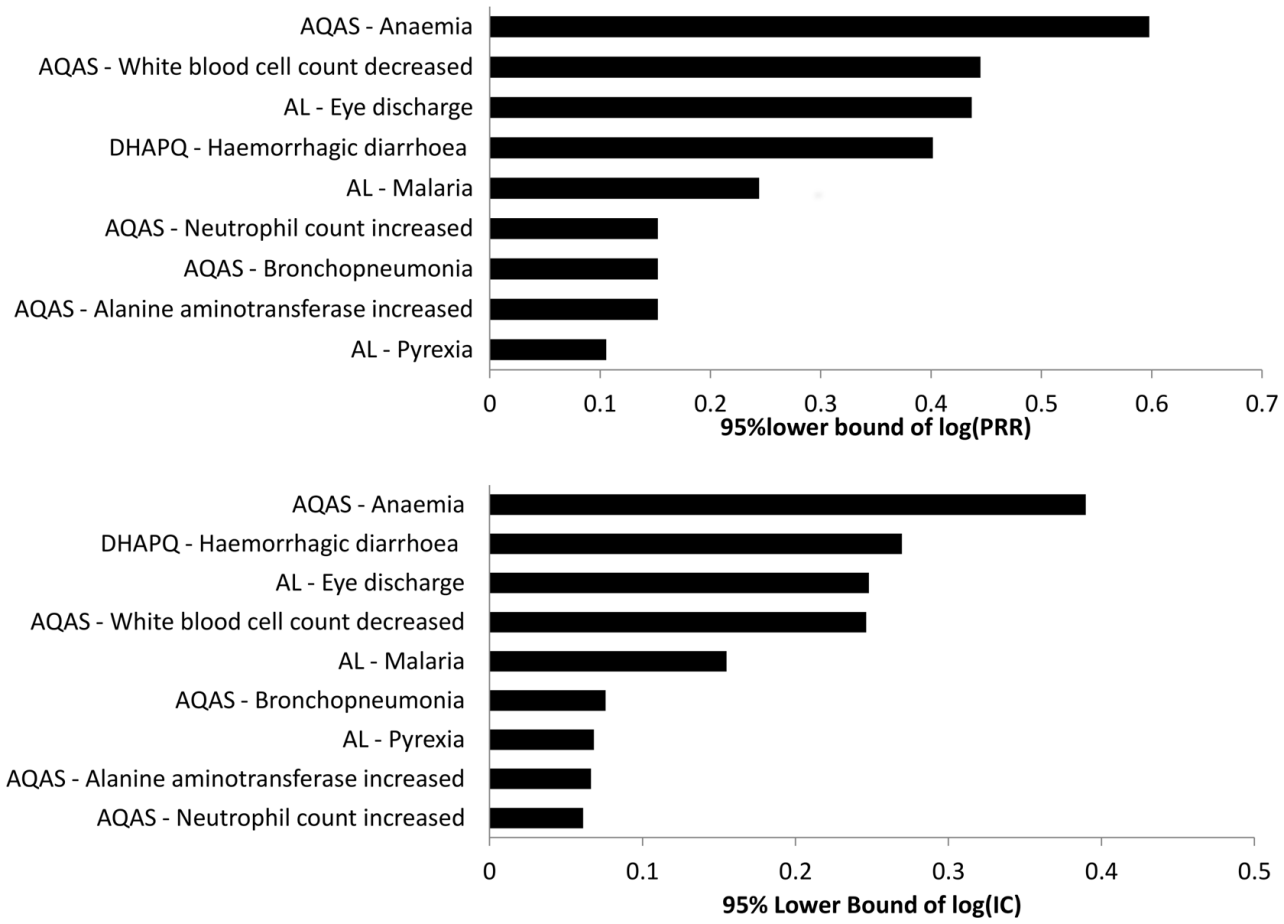
Ranking of drug-event combinations according to the signal index of the PRR (Upper panel) and BCPNN (Lower panel) methods.

Both DMAs used in this analysis produced similar signals with similar frequency suggesting they were of comparable performance for this dataset. There was a slight difference in the ranking done on the lower bound of the confidence interval that takes into account the variance of the disproportionality measure of association. Suspected signals ASAQ-Anaemia, ASAQ- increased Alanine amino Transferase, AL-Malaria and AL-Eye discharge had the same ranking by both methods (Figure 1) according to the respective method’s signal index. A drug-event combination like ‘AL-Pyrexia’ had many reports in the database though it did not translate into higher signal index and was ranked lowest by PRR though other combinations like ‘AL-eye discharge’ had fewer reports in the database.

## Discussion

Each of the two DMAs used a different method of calculating the signal index and the ranking done on the lower bound of confidence interval of drug-AE signal indices differed. Both algorithms produced equal number of signals, suggesting that for this dataset their performance was comparable.

A drug-event combination like ‘AL-Pyrexia’ had many reports in the database but did not translate into higher signal index and hence ranked lower than other combinations with fewer reports like ‘AL-eye discharge’. This could be due to the fact that similar AEs occurred among the other drugs of interest. Additionally, some conditions in the signalled drug-event combinations were related to each other and therefore removing one combination affected the appearance of the other as a signal, e.g. ‘bronchopneumonia’ and ‘neutrophil count increased’ (12 reports each); or malaria with pyrexia.

This paper looks at paediatric pharmacovigilance, an area in drug safety research that has not gained momentum especially in the Sub-Saharan African region. This is true for paediatric pharmacovigilance in both pre-market and post-market phases of drug evaluation. It is vital to develop effective methods for early detection of ADRs and drug safety epidemiological studies in children [21]. In Africa, the need to improve pharmacovigilance methods applies to both new and existing drugs that have been used for many years in children. Indeed, the pharmacokinetics of a given drug may be altered in paediatric patients consequent to intrinsic (e.g. gender, genotype, ethnicity) and/or extrinsic (e.g. acquired disease states, diet) factors which may occur in the first two decades of life. Overall, there is limited data available on safety in children during the drug development process as children are excluded from most randomized clinical trials. Moreover, in clinical practice some significant AEs may not be recognised and documented because paediatric patients cannot communicate discomfort associated with the drug. Neonates and infants cannot communicate meaning that the detection of ADRs requires a kin mother/care taker, paediatrician or a laboratory test.

The signal detection process using phase IIIb/IV clinical studies data illustrates the potential utility of this approach for future drug monitoring, especially in Africa where many clinical studies are conducted and safety data are usually archived after producing the trial report. Clinical trials databases are usually well designed, of high quality and high level of completeness for clinical information and hence are more effective for signal detection. Indeed the use of spontaneously reported AE data have been associated with a number of limitations when analysing with the inter-product quantitative DMA such as underreporting, lack of precision in the AE definition, uncertainty in estimating the extent of the drug-exposed population, unreliability due to the highly variable quality of the reports [22], [23]. Even with such limitations, DMA continue to be a useful pharmacovigilance tools, utilised by international organisations and regulatory authorities that possess large spontaneously reported AE databases which routinely generate and monitor signals.

The use of clinical studies databases would offer an ideal complement to spontaneous reporting systems (SRS), since such databases do not suffer the caveats of the SRS and could facilitate a rapid identification of new signals. Data mining has been used to explore cardiovascular clinical trials databases [24], and other specialist databases like the US Vaccine Adverse Event Reporting System [25]. Clinical trial data have typically not been amenable to safety data mining techniques because of the small sample sizes and narrow variations in database designs. However, their high intrinsic quality and completeness in clinical information make them invaluable sources of information. On the other hand the small sample size setback can be averted by pooling of safety databases from different multi-country, multi-site studies as was the case for the present study.

Data mining algorithms (DMAs) for quantitative signal detection are practical on large, late stage trials or pooled studies, though screening for signals ought to begin early in the process. After identifying safety signals or areas of interest, they can be tracked over time and lead to further actions. Standardised documentation provides evidence of continuous proactive vigilance and data can be compared to spontaneous reports and observational data.

One limitation of DMAs is the potential variation in statistical properties of the standard signalling threshold when applied to different datasets. Different thresholds may be needed for more common *versus* rare outcomes/events which is not yet currently done in practice. Another limitation is that a strong association between an adverse reaction and a drug that is not of specific interest (e.g. for large values of ‘b’ in Table 1) may reduce the likelihood of detecting a true signal between that adverse event and the drug of interest. This situation could occur when a drug that is an indication for the adverse event of interest is included in the dataset (and thus included in the ‘all other drugs’ comparison group).

A specific limitation to the PRR method is the impossibility of calculating the PRR estimate in the absence of adverse events of interest reported for the comparison drug (s) (i.e., if b = 0 in Table 1). Further, since the PRR is a ratio of two proportions, its value is unstable for small sample sizes as it is the case for most clinical trials.

The methods used in this analysis worked well in the identification of single drug-event signals but are not suitable for the identification of drug interactions since they consider only one drug at a time. Other methods such as association rules (a priori algorithms) and clustering algorithms are more appropriate for the identification of drug interactions. Association rules approach was used by Harpaz et al [26] using data from FDA spontaneous adverse events reporting system which demonstrated the opportunity to develop and use novel algorithms to detect drug interactions.

The signals evaluation method in this analysis was adopted because there is no structured database of possible ADRs in Africa other than the VigiBase which is hosted at the WHO safety monitoring centre in Uppsala [27]. Such a database would considerably improve the efficiency of signal detection process by allowing for filtering or flagging reaction reports of previously unknown reactions. Only five signals remained after the evaluation process that involved use of manufacturers’ package leaflets, a Multi-Drug Symptom/Interaction Checker (DoubleCheckMD) and experts’ opinions. These are regarded as new hypotheses that may need further studying in pharmacoepidemiological studies.

In conclusion, this paper has demonstrated the possibility of utilising data mining algorithms with clinical studies safety data for key pharmacovigilance activities like signal generation and evaluation especially when datasets from multi-centre clinical studies are pooled. These methods can be used to facilitate the initial signal monitoring steps of “signal strengthening”, “signal follow-up” and also for hypothesis generation before the implementation of epidemiological and experimental studies. This study also contributes to the limited but important literature on paediatric pharmacovigilance, especially in Sub-Saharan Africa.
